# Sex-Specific Impact of Pain Severity, Insomnia, and Psychosocial Factors on Disability due to Spinal Degenerative Disease

**DOI:** 10.1155/2020/8496527

**Published:** 2020-05-06

**Authors:** Keito Koh, Keiko Yamada, Tatsuya Enomoto, Aiko Kawai, Saeko Hamaoka, Satoko Chiba, Masako Iseki

**Affiliations:** ^1^Department of Anesthesiology and Pain Medicine, Juntendo University Shizuoka Hospital, Izunokuni 410-2211, Japan; ^2^Department of Anesthesiology and Pain Medicine, Juntendo University Faculty of Medicine, Tokyo 113-8431, Japan; ^3^Department of Psychology, McGill University, Motreal H3A 1G1, Canada

## Abstract

**Purpose:**

Pain experience due to spinal degenerative disease decreases activity of daily living and quality of life. The present cross-sectional study was aimed at examining the sex-specific impact of pain severity, psychosocial factors, and insomnia on the disability due to chronic pain arising from spinal degenerative disease.

**Methods:**

In total, 111 outpatients with chronic spinal degenerative on initial diagnosis were analyzed. The definition of chronic spinal degenerative disease was (1) pain duration ≥3 months, (2) findings of nerve root compression on neurological examination and imaging, and (3) localized neck or lower back pain (not widespread, upper or lower limb pain). We used Numerical Rating Scale (NRS), Pain Disability Assessment Scale (PDAS), Hospital Anxiety and Depression Scale (HADS), Pain Catastrophizing Scale (PCS), and Athens Insomnia Scale (AIS) to assess patients. Univariate regression analysis was performed to investigate whether sex influences the PDAS score, and sex-stratified multivariate regression analysis was conducted to identify the variables associated with the PDAS score.

**Results:**

Sex was identified as a predictor of the PDAS score (standardized coefficient (*β*) = 0.28; 95% confidence interval (CI), 0.10–0.46; *p*=0.003). In men, the AIS score was associated with PDAS (*β* = 0.36, 95% CI 0.09–0.63). Age (*β* = 0.31, 95% CI 0.06–0.55) and NRS (*β* = 0.40, 95% CI 0.14–0.67) were associated with PDAS in women. HADS-A, HADS-D, and PCS were not associated with PDAS in both sexes.

**Conclusion:**

Insomnia was associated with disability in men, whereas aging and pain severity were associated with disability in women. Catastrophic thinking was not associated with disability in both sexes.

## 1. Introduction

Chronic pain arises due to various diseases, including musculoskeletal disorders, postherpetic neuralgia, and fibromyalgia, and the resulting disability, which impedes the performance of daily activities, is an important issue. Suggestions for the treatment and management of chronic pain emphasize on reducing pain-related disabilities despite presence of persistent pain, rather than its complete elimination. Thomas and Lee addressed that “zero pain is not the goal.” [1] Multidisciplinary approach includes physical therapy and cognitive behavioral therapy to treat patients with chronic pain target to improve disability due to pain [2].

Particularly, pain arising from musculoskeletal disorder causes disability owing to its involvement in performing physical activity. Chronic pain due to aging-related decline in musculoskeletal functions is defined as “locomotive pain,” and it decreases activity of daily living and quality of life among the old people [3]. The increasing number of patients with locomotive pain, in addition to the increasing medical cost for the management of old people, adds to social burden [4].

Musculoskeletal pain majorly involves the lower back and neck, and it originates from the intervertebral discs of the spine [5]; musculoskeletal pain is a major symptom related to disability and impairment in Quality of Life (QOL) in the great burden of disease (GBD) study conducted in 2015 [6]. In the GBD ranking, lower back pain ranks first followed by neck pain at fourth, whereas major depressive disorder ranks second [6]. Additionally, the lifetime prevalence of spinal diseases is more than 50% in both developing and developed countries [7, 8]. Sustaining a high QOL requires the treatment and prevention of disability due to pain arising from degenerated intervertebral discs of the spine. A higher prevalence of spinal degenerative disease than spinal inflammatory disorder was reported for spinal diseases [9]. In spinal degenerative disease, the transformed intervertebral disk in the spine presses against the motor and sensory nerve, leading to motor nerve impairment and pain. For severe motor nerve impairment in the upper and lower limbs, surgery is often preferred to reduce the pressure by the transformed spine and intervertebral disk [10]. Less severe cases without motor nerve impairment in the upper and lower limbs are often resolved by analgesics, without the need for surgery [10]. However, those patients report severe disability although nerve compression by the transformed spine is unremarkable. The reason for this discrepancy between clinical diagnosis by health care providers and self-reported disability among patients needs further investigation.

Recently, health care providers have been suggested to consider patients' sex and gender differences for managing pain [11]. Generally, the prevalence of chronic pain is higher in women than in men [12], owing to differences in biological factors such as hormones and social factors [13]. Sex difference in disability among depression patients has been reported in a previous study which observed the association of the severity of anxiety and insomnia with disability. In patients with musculoskeletal pain, including chronic spinal disease, pelvic pain, and knee disease-associated pain, reports suggest that the disability is more severe in women than men [14]. However, more studies are required to delineate the differences in sex on the disability arising due to chronic musculoskeletal pain.

The present study was aimed at examining the sex-specific impact of pain severity, psychosocial factors (e.g., depression, anxiety, and catastrophizing), and insomnia on the disability due to chronic musculoskeletal pain arising from spinal degenerative disease.

## 2. Methods

This was a cross-sectional, retrospective study.

### 2.1. Ethical Considerations

The Institutional Review Board of Juntendo University Hospital approved the study protocol (no. 19-044). The requirement for informed consent from participants was waived because of the retrospective study design. Furthermore, the present retrospective study was opted out from the requirement of informed consent on the website of Juntendo University Hospital.

### 2.2. Study Population

Among 122 outpatients with chronic spinal degenerative disease on initial examination in 2016, 11 outpatients with missing values in the self-reported questionnaire were excluded. Chronic spinal degenerative disease was defined as (1) persistent pain for more than three months [15], (2) findings of nerve root compression because of the degenerative spine or the intervertebral disc on neurological examination and magnetic resonance imaging but without surgical indication, and (3) localized neck or lower back pain (but not widespread, upper or lower limb pain).

The chronic spinal degenerative diseases are categorized as lumbar or cervical diseases. Lumbar spinal canal stenosis, degenerative spondylolisthesis, and herniated lumbar intervertebral disk include the lumbar diseases, whereas cervical spondylotic myelopathy due to degeneration of the cervical spine, cervical radiculopathy, and herniated cervical intervertebral disk comprise the cervical diseases.

### 2.3. Measurement

#### 2.3.1. Pain Severity

The maximum pain intensity was evaluated for 24 hours using Numerical Rating Scale (NRS) [16] and comprised scores ranging from 0 (“no pain”) to 10 (“worst pain”).

#### 2.3.2. Pain-Related Disability

We assessed the disability in performing 20 daily activities during the past week using Pain Disability Assessment Scale (PDAS). The PDAS is a Likert scale from 0 (“pain did not interfere with this activity”) to 4 (“pain completely interfered with this activity”) and total scores ranging from 0 to 60. A high PDAS score indicated a high degree of disability [17]. A previous clinical study in Japanese patients with chronic low back pain reported the mean value of the PDAS score to be 29.8 (standard deviation (SD), 15.3) [18].

#### 2.3.3. Hospital Anxiety and Depression Scale

Anxiety and depression were evaluated by Hospital Anxiety and Depression Scale-anxiety (HADS-A) and HADS-depression (HADS-D), respectively [19]. HADS-A and HADS-D consist of seven items each, and the score for each item ranges from 0 to 3; the total score can range from 0 to 21. A previous study reported that 8 points or more on the HADS scale are indicative of anxiety or depression [20].

#### 2.3.4. Pain Catastrophizing Scale

Pain Catastrophizing Scale (PCS) evaluates a catastrophic thought [21] and consists of 13 items with scores ranging from 0 to 4 points, and the total score can range from 0 to 52. A high PCS score is indicative of a higher catastrophic thought. We considered a score of 30 points or more as indicative of a strong catastrophic thought. A PCS score of more than 30 corresponds to the 75th percentile of the distribution of PCS scores in clinic samples of chronic pain patients. The mean value of the PCS score among Japanese patients with chronic low back pain was 35.1 (SD, 12.9) [18].

#### 2.3.5. Insomnia

Insomnia was evaluated by the Athens Insomnia Scale (AIS) [22]. The AIS evaluates sleep during the past month using 8 items, and each item is rated on a 4-point Likert scale from 0 to 3. Chronic pain patients with AIS ≥8 points were clinically defined as having insomnia [23].

### 2.4. Statistical Analyses

First, we used univariate regression analysis to examine whether sex contributed to disability due to pain. Next, sex-stratified multivariate regression analysis was conducted to identify the variables associated with disability. Age, body mass index (BMI), pain duration, NRS, PCS, HADS-A, HADS-D, and AIS were considered as explanatory variables.

## 3. Results

The proportion and means of the characteristics used are shown in Table 1. Among the 111 patients, 61 were men (55%), and 50 were women (45%). The women were old and frail; experienced shorter duration of pain; and presented a higher proportion of disability, anxiety, and depression than men did.

The univariate regression analysis identified sex as a predictor of the PDAS score (standardized coefficient (*β*) = 0.28; 95% confidence interval (CI), 0.10–0.46; *p*=0.003; data not shown in the tables).

Table 2 indicates the association of age, BMI, pain duration, pain intensity, and psychosocial factors with disability. In men, the AIS score was associated with PDAS (*β* = 0.36, 95% CI 0.09–0.63). Age (*β* = 0.31, 95% CI 0.06–0.55) and NRS (*β* = 0.40, 95% CI 0.14–0.67) were associated with PDAS in women. HADS-A, HADS-D, and PCS were not associated with PDAS in both sexes.

## 4. Discussion

The present study revealed that factors that influenced pain-related disability were different in both sexes with spinal degenerative disease. In men, insomnia was significantly associated with disability, whereas in women, age and pain intensity were significantly associated with disability. Catastrophizing, anxiety, and depression were not associated with pain-related disability in both sexes.

A previous study described that pain severity had a stronger association with disability in women than in men [11]. Another study reported no sex differences in the association between pain severity and disability [24], revealing an inconsistency in results. This may be because of differences in the study settings as (1) causes of pain were different in each study and (2) some studies used patient data after intervention [25, 26]. In addition, treatment by a previous physician could be a confounding factor.

Although previous studies targeted a broad interpretation of chronic musculoskeletal disease, the study is the first to focus on specific chronic musculoskeletal diseases (e.g., spinal degenerative diseases) to examine the sex-specific impact of various factors on pain-related disability.

Insomnia affected disability induced by chronic pain because of spinal degenerative disease only in men. A previous study has reported an association between insomnia and chronic pain due to spinal degenerative disease [27]. Although few previous studies have reported the association between insomnia and disability due to chronic pain [28, 29], they did not report any sex differences. With respect to depression, which is often complicated with chronic pain, insomnia was associated with disability only in men [29]; however, the underlying mechanism is not clearly indicated [30]. A potential mechanism for the association between insomnia and disability in men may be the fact that the prevalence of insomnia related to the obstructive sleep apnea symptom (OSAS) is higher in men than in women [31]. Patients with OSAS are well known to experience disability in the daytime even if they do not have chronic pain [32]. Therefore, daytime disability would be higher among chronic pain patients with OSAS-related insomnia than among chronic pain patients without insomnia. Thus, OSAS is a potential confounding factor between insomnia and disability among chronic pain patients. In the current study, the means of BMI were higher in men than in women, and in general, OSAS is often caused by obesity. Thus, OSAS may be a potential confounding factor between insomnia and disability among chronic pain patients. However, we did not examine the incidence of OSAS, and future examination is necessary to confirm whether OSAS is a potential confounding factor between insomnia and disability among chronic pain patients.

On the contrary, age was significantly associated with disability in women. In brief, disability in women with chronic pain due to spinal degenerative disease increases with age. An age-related decrease in skeletal muscle mass was observed in women than in men older than 60 years, and disability by aging was more remarkable in women than in men [33]. This may be a potential mechanism underlying the association between aging and disability in women. However, this is a speculation because in this study, we did not measure skeletal muscle mass of men and women.

Notably, catastrophizing, anxiety, and depression were not associated with disability in both sexes. These results were not consistent with those of previous studies in which psychosocial factors, such as catastrophizing, anxiety, and depression, were associated with chronicity of pain [32]. The present study examined patients with chronic spinal degenerative localized neck or lower back pain (not upper or lower limb pain), who were likely to have nociceptive pain but less likely to have neuropathic pain associated with advanced spinal degenerative disease. A previously published prospective multicenter study reported that disability due to neuropathic pain was associated with pain catastrophizing [34]. However, patients with nociceptive pain may not be associated with catastrophic thinking, anxiety, and depression despite three or more months of pain duration. Furthermore, the low prevalence of neuropathic pain would consequently induce lower levels of anxiety and depression in the current study; however, this result was inconsistent with that published previously. Indeed, the mean HADS score for anxiety and depression was 7.0 and 8.4 points, respectively, in women and 6.2 and 6.9, respectively, points in men, which are lower than the cutoff score of HADS (8 points).

In contrast, catastrophizing was severe in the current study; the mean PCS score was more than 30 points for both men and women [21]. Among patients with focal lesions (e.g., nerve compression because of spinal degenerative disease), which clearly cause pain, catastrophizing may have a less effect on disability even if they have pain during over 3 months, although higher catastrophizing, anxiety, and depression were reported in patients with “nonspecific” low back pain in a previous study [35].

There are a few limitations in our study. First, we did not examine the presence of female-specific diseases. Menstruation-related conditions such as premenstrual syndrome, menstrual pain, delivery, and menopause may act as confounders for the sex-specific impact of disability due to pain. Additionally, women showed a higher prevalence of chronic pain owing to fibromyalgia and migraine compared with men [11]. Such female-specific health conditions may aggravate the disability due to pain. Therefore, it will be necessary to examine, particularly, the diseases specific to women and their health conditions in future when we examine the sex differences of disability on daily activities due to the chronic pain. Second, we did not stratify patients who visited a primary health care facility and those who had visited one or more clinics/hospitals previously. Although the latter patients received previous treatment at health care facilities, which might have affected patient evaluation, we did not examine the effect of previous treatment in this study. Third, the present study entails a cross-sectional design, and hence, we were unable to examine whether the interventions for insomnia in men and pain intensity in women improve disability. Interventional studies need to be conducted in the future. Finally, the present study was conducted in a single institution comprising a small sample size and, hence, is not representative for patients with spinal degenerative disease. Large-scale studies with patient data from multiple institutions are, therefore, necessary in the future.

In conclusion, we observed sex differences in disability due to pain caused by spinal degenerative disease. Insomnia was associated with disability in men, whereas aging and pain severity were associated with disability in women. Although catastrophic thinking was severe, anxiety and depression were not severe among both men and women for chronic pain due to spinal degenerative disease. Considering sex/gender differences in disability is important for treatment patients with pain caused by spinal degenerative disease.

## Figures and Tables

**Table 1:** Mean values and proportions of characteristics (*n* = 111).

	Men, *n* = 61		Women, *n* = 50	
	*n*	(%)	*n*	(%)
Pain site				
Neck pain with cervical vertebral degenerative	16	(26.2)	14	(28.0)
Low back pain with lumbar degenerative	45	(73.8)	36	(72.0)
	Means	SD	Means	SD
Age, year	60.1	15.2	64.6	14
BMI, kg/m^2^	24.1	2.7	21.5	2.6
Pain duration, months	52.5	65.4	37.2	45.5
NRS (0–10)	6.7	2.3	6.8	2.3
PDAS (0–60)	22.3	13.1	30.1	13.4
HADS anxiety (0–21)	6.2	4.1	7.0	4.4
HADS depression (0–21)	6.9	4.3	8.4	5.4
PCS (0–52)	32.3	10.8	32.1	10.4
AIS (0–24)	7.6	4.6	7.7	4.8

SD: standard deviation, BMI: body mass index, NRS: Numerical Rating Scale, PDAS: Pain Disability Assessment Scale, HADS: Hospital Anxiety and Depression Scale, PCS: Pain Catastrophizing Scale, and AIS; Athens Insomnia Scale.

**Table 2:** Multiple regression analysis examining the association between sample characteristics and pain disability (*n* = 111).

	Men, *n* = 61	Women, *n* = 50
Dependent = PDAS	*β* (95% CI)	*β* (95% CI)
	(*R*^2^ = 0.44, F_change_ 5.8)	(*R*^2^ = 0.44, F_change_ 4.1)
Age	0.01 (−0.20–0.23)	0.31 (0.06–0.55)^*∗*^
BMI	−0.04 (−0.26–0.18)	−0.14 (−0.41–0.12)
Pain duration	0.03 (−0.19–0.25)	−0.16 (−0.43–0.10)
NRS maximum	0.20 (−0.03–0.43)	0.40 (0.14–0.67)^*∗∗∗*^
PCS	0.18 (−0.08–0.44)	0.21 (−0.13–0.56)
HADS anxiety	−0.01 (−0.39–0.38)	0.08 (−0.34–0.49)
HADS depression	0.14 (−0.22–0.51)	−0.10 (−0.48–0.28)
AIS	0.36 (0.09–0.63)^*∗*^	0.13 (−0.20–0.45)

Multiple regression analysis was performed: ^*∗*^*p* < 0.05, ^*∗∗∗*^*p* < 0.001. PDAS: Pain Disability Assessment Scale, BMI: body mass index, NRS: Numerical Rating Scale, PCS: Pain Catastrophizing Score, HADS: Hospital Anxiety and Depression Scale, AIS: Athens Insomnia Scale, *β*: standardized regression coefficient, and CI: confidence interval.

## Data Availability

Data cannot be shared publicly because data contain potentially identifying or sensitive patient information. Based on regulations for ethical guidelines in Japan, the Institutional Review Board for Clinical Research of Juntendo University Hospital imposed restrictions on the data collected in this study. All enquiries should be addressed to the Data Management Committee via e-mail: kenkyu5858@juntendo.ac.jp.
